# Performances and determinants of proficiency testing in clinical laboratory services at comprehensive specialized hospitals, northwest Ethiopia

**DOI:** 10.1038/s41598-024-58525-6

**Published:** 2024-04-02

**Authors:** Negesse Cherie, Teshiwal Deress, Maereg Wolde, Bisrat Birke Teketelew, Mebratu Tamir, Abiy Ayele Angelo, Amare Mekuanint Terekegne, Elias Chane, Mesele Nigus, Dereje Mengesha Berta, Kasaw Adane

**Affiliations:** 1 Department of Quality Assurance and Laboratory Management, School of Biomedical and Laboratory Sciences, College of Medicine and Health Sciences, University of Gondar, Gondar, Ethiopia; 2 Department of Health Promotion and Health Behavior, College of Medicine and Health Science, University of Gondar, Gondar, Ethiopia; 3 Department of Hematology and Immunohematology, School of Biomedical and Laboratory Sciences, College of Medicine and Health Sciences, University of Gondar, Gondar, Ethiopia; 4 Department of Medical Parasitology, School of Biomedical and Laboratory Sciences, College of Medicine and Health Sciences, University of Gondar, Gondar, Ethiopia; 5 Department of Immunology and Molecular Biology, School of Biomedical and Laboratory Sciences, College of Medicine and Health Sciences, University of Gondar, Gondar, Ethiopia; 6 Department of Clinical Chemistry, School of Biomedical and Laboratory Sciences, College of Medicine and Health Sciences, University of Gondar, Gondar, Ethiopia

**Keywords:** Health care, Health occupations

## Abstract

Proficiency testing (PT) is an impartial laboratory performance-evaluating system using an independent body. It is a mandatory accreditation requirement and means for improving the laboratory’s performance. The study aimed to evaluate the performance of PT, with a focus on identifying and discussing determinants that influence PT performance at comprehensive specialized hospitals in northwest Ethiopia. A retrospective cross-sectional study was carried out from 2020 to 2022. Using a convenient sampling technique, laboratory tests with recorded PT results in each hospital laboratory were included. A data collection template and customized checklists were used to collect the data. Epi Data Version 3.1 for data entry and STATA Version 14.1 for cleaning and analysis were used. Binary logistic regression analyses were used. Variables with p < 0.05 in the multivariable logistic regression were considered to be statistically significant. Over nine cycles, 3807 PT challenges were distributed. The total failure rate of the laboratories was 32.4%, with a peak failure rate of 40.3% in 2020, after which the failure rate was decline to 20.6% in 2022. Among the five laboratory sections, molecular biology had the lowest failure rate (22.2%), while microbiology had the highest failure rate (56.5%). Multivariate logistic regression revealed that PT results reported without appropriate unit of measurement (AOR 7.5), lack of corrective action for PT nonconformance (AOR 7.1), and reagent unavailability (AOR 6.1) had significant effects on PT performance (p < 0.001). The results of this study showed that the overall performance of the laboratory was lower. Reporting PT results without appropriate units of measurement and not taking corrective action for PT nonconformance were the major aggravating factors for high failure rates.

## Introduction

The implementation of corrective measures, quality indicators, Internal Quality Control (IQC), and External Quality Assessment (EQA) are tools for reducing laboratory errors and improving quality in laboratory medicine. Among these tools, the EQA and IQC have been extensively used to reduce analytical errors. Moreover, beyond the IQC process, Proficiency Testing (PT) is vital for identifying routine analytical errors using an external agency^1^. As a result, laboratories must participate in proficiency testing programs as part of routine quality assurance^2^.

Proficiency testing involves the use of unknown specimens obtained from an external source as a means of external quality and accuracy assessment^3^. In specimen management, equipment operation, and result reporting, an essential comparative and objective evaluation approach is provided for the laboratory^4^. Participation in PT is a requirement for accreditation and a way to increase the performance of the laboratory^5,6^.

Under the Clinical Laboratory Improvement Amendment (CLIA) of 1988, clinical laboratories conducting nonwaived tests are required to conduct proficiency testing^3^. It can be a useful tool for quality improvement and is a crucial part of a laboratory quality management system^7^. To provide accurate analytical results, laboratories must effectively participate in the PT program, which assesses the laboratory’s analytical performance in comparison to its peers or reference standards^8^. It is also an invaluable tool for evaluating laboratory performance and increasing the precision and dependability of test findings^9^.

To maintain trust in the caliber of laboratory performance, PT is crucial for providing staff competencies, training and educating testing workers, assessing novel techniques or technologies, and comparing test findings with those from other comparable instruments^9^. Effective participation of laboratories in the PT program can uncover 48%, 86%, and 76% for the preanalytical, analytical, and postanalytical errors, respectively^10,11^.

However, there is a dearth of published information regarding PT performance and its determinants in clinical laboratories in the northwest region of Ethiopia. As a result, the study aimed to pinpoint and clarify the PT performance of the laboratories and any potential difficulties for those laboratories to encounter during the participation and implementation of PT. In addition, it is vital to inform policy makers about the status of laboratory PT performance to make appropriate interventions for the better performance and continuous quality improvement. The study also encouraged the participating laboratories to establish a standardized system for the implementation of PT.

## Materials and methods

### Study design and setting

A retrospective cross-sectional study was conducted at comprehensive specialized hospital laboratories in northwest Ethiopia from 2020 to 2022. There are three regional and two federal comprehensive specialized hospitals in the area^12,13^. The study was conducted in one federal (University of Gondar) and two regional (Debre Markos and Felege Hiwot) comprehensive specialized hospitals. Among these hospital laboratories, a total of 198 laboratory professionals served clinical laboratory services in the hematology, parasitology, clinical chemistry, microbiology, and molecular biology sections. These hospital laboratories access PT samples three times per year from an international organization called One World Accuracy (digital PT program) through Ethiopia and Amhara public health institutes via postal services.

### Eligibility criteria

The study included routinely performed laboratory tests, in which PT data were recorded in all three hospital laboratories. Laboratory tests with incomplete PT data for the dependent and/or independent variables were excluded from the study.

### Sample size and sampling technique

The data on the PT sample results for 30 routinely performed laboratory tests from 2020 to 2022 were collected. A convenience sampling technique was used to select laboratory test results from among the participating hospital laboratories.

### Data collection tools and procedures

Retrospective data from nine PT cycles (2020–2022) were collected by using a pretested data collection template and customized checklists, which were adopted from CLIA standard guidelines^14^. Performance scores were collected using the data collection template, which was completed from PT feedback that was sent to each participating laboratory. The performance scores of each analyte in each cycle were extracted as acceptable (ACC) for those that met the CLIA standard requirement (80%) and above and unacceptable (UNACC) for those that had a score less than 80%. Overall performance was calculated by summing all grading area responses with acceptable and unacceptable scores. Laboratories that met 80% and above were interpreted as having a good analytical performance score. Data related to our key independent variables were collected through customized checklists from the PT feedback report and the laboratory logbook. One data collector per hospital with a background in the laboratory profession was deployed to collect the data.

### Data analysis and interpretation

The data were coded and entered into Epi Data version 3.1 software and subsequently exported to STATA version 14.1 for analysis. Descriptive statistics such as frequencies and percentages were calculated. A chi-square test for model assumptions and variable associations with the dependent variable was performed. To measure the association between the dependent and independent variables, bivariate and multivariable binary logistic regression were used. Variables with p values less than 0.25 were fitted to the multivariable logistic regression analysis to control for possible confounding effects. Both the crude (COR) and adjusted odds ratio (AOR) with the corresponding 95% confidence interval (CI) were calculated to measure the strength of the association. Finally, variables with p values less than 0.05 were considered to be significantly different.

### Ethical consideration

Ethical clearance was obtained from Ethical Review Committee of the School of Biomedical and Laboratory Sciences (Ref: SBMLS/521), College of Medicine and Health Sciences, University of Gondar. The support letter was taken to the referral hospitals, and permission was obtained from the hospital manager, diagnostic laboratory coordinator, and laboratory section heads. After a clear explanation of the purpose and benefits of the study, written informed consent was obtained from the study participants. To ensure confidentiality, all study participants’ result and information were kept coded, and personal identifiers were removed and analyzed anonymously. Participation in the study was entirely voluntary and study participants had the chance to refuse from being in the study.

### Declarations

All procedures were performed in accordance with relevant guidelines’ in the manuscript.

## Results

In this study, one federal and two regional comprehensive specialized hospital laboratories participated in all nine cycles from 2020 to 2022. A total of 3807 PT challenges were enrolled for 30 routinely performed laboratory test parameters. There were five PT challenges for each test parameter, except for CD_4_, blood parasite identification, and gene expert (two PT challenges).

### Analytical performance of proficiency testing

The overall acceptable PT performance of the laboratories was 2573 (67.6%), with a failure rate of 1234 (32.4%). In 2020, the lowest PT performance was observed with the highest failure rate of 511 (40.3%), while in 2022, the best PT performance was observed with the lowest failure rate of 262 (20.6%). The overall failure rate gradually decreased from 511 (40.3%) in 2020 to 461 (36.3%) in 2021 to 262 (20.6%) in 2022 (Table 1). For more information see Table S1.

**Table 1:** Overall performance of proficiency testing in the 3 years in each section of the laboratory.

Laboratory sections	Performances	Year							
		2020		2021		2022		Total	
		#	%	#	%	#	%	#	%
Chemistry	ACC	272	50.4	355	65.7	390	72.2	1017	62.8
	UNACC	268	49.6	185	34.3	150	27.8	603	37.2
Hematology	ACC	303	67.3	259	57.6	394	87.6	956	70.8
	UNACC	147	32.7	191	42.4	56	12.4	394	29.2
Microbiology	ACC	123	62.1	135	68.2	157	79.3	415	43.5
	UNACC	75	37.9	63	31.8	41	20.7	179	56.5
Molecular biology	ACC	48	76.2	48	76.2	51	81	147	77.8
	UNACC	15	23.8	15	23.8	12	19	42	22.2
Parasitology	ACC	12	66.7	11	61.1	15	83.3	38	70.4
	UNACC	6	33.3	7	38.9	3	16.7	16	29.6
Total	**ACC**	758	59.7	808	63.7	1007	79.4	**2573**	**67.6**
	**UNACC**	511	40.3	461	36.3	262	20.6	**1234**	**32.4**

*ACC* acceptable, *UNACC* unacceptable, *#* frequency, *%* percentage.

Significant values are in bold.

### Trend of proficiency testing performance in the three consecutive years at each section of the laboratory

**Figure 1:**
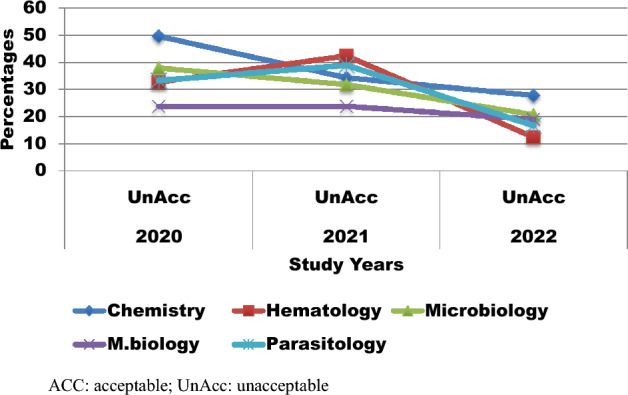
Trend of proficiency testing performance over 3 years (2020–2023) of study at comprehensive specialized hospitals, Northwest Ethiopia, 2023. *ACC* acceptable, *UnAcc* unacceptable.

Among the five laboratory sections, the highest failure rate was recorded in microbiology, accounting for 179 (56.5%) specimens. However, according to molecular biology, the data showed a relatively good analytical PT performance score, with the lowest failure rate of 42 (22.2%). Except for the hematology and parasitology sections of the laboratory, the overall failure rate gradually decreased each consecutive year (Fig. 1).

### Determinants associated with the PT performance scores of analytes

In the present study, multivariable logistic regression showed that equipment failure and downtime (AOR 2.6, 95% CI (1.05–6.44); unavailability of adequate reagents and supplies (AOR 6.1, 95% CI (2.58–14.77); lack of daily IQC practice (AOR 3.8, 95% CI (1.43–10.30); PT result without appropriate unit of measurement (AOR 7.5, 95% CI (3.06–18.57)); and lack of corrective actions taken for PT nonconformance (AOR 7.1, 95% CI 6.8 (2.99–16.87)) were significant determinants that affected the PT performance of the analytes (Table 2).

**Table 2:** Determinants contributing to proficiency testing performance at comprehensive specialized hospitals, northwest Ethiopia, 2023.

Variables		PT performances				COR 95% CI	AOR 95% CI	p value
		Acceptable		Unacceptable				
		#	%	#	%			
PT sample tested with properly functional equipment	Yes	57	21.1	33	12.2	1	1	0.038*
	No	30	11.1	150	55.6	8.6 (4.83 –15.43)	2.6 (1.05–6.44)	
PT sample performed with adequate reagent	Yes	64	23.7	49	18.1	1	1	< 0.001*
	No	23	8.5	134	49.6	7.6 (4.26–13.56)	6.1 (2.58–14.77)	
PT sample not performed due to reagent stockout	Yes	47	17.4	33	12.2	1	1	0.783
	No	40	14.8	150	55.6	5.3 (3.03–9.40)	1.1 (0.41–3.18)	
Does the laboratory perform daily IQC practice	Yes	43	15.9	48	17.8	1	1	0.007*
	No	44	16.3	135	50	2.7 (1.61–4.68)	3.8 (1.43–10.30)	
Is quality manual available in the laboratory	Yes	58	21.5	93	34.4	1	1	0.095
	No	29	10.7	90	33.3	3.9 (2.21–7.03)	2.1 (0.87–5.36)	
Is PT sample tested by the same staff performing routine patient testing	Yes	25	9.3	16	5.9	1	1	0.731
	No	62	23.0	167	61.9	4.2 (2.10–8.40)	1.2 (0.31–5.27)	
Does the laboratory report PT result with reference range	Yes	45	16.7	36	13.3	1	1	0.374
	No	42	15.6	147	54.4	4.4 (2.50 -7.63)	1.6 (0.55–4.76)	
Does the laboratory report PT result with appropriate unit of measurement	Yes	66	24.4	31	11.5	1	1	< 0.001*
	No	21	7.8	152	56.3	15.4 (8.25–28.8)	7.5 (3.06–18.57)	
Does laboratory recorded and document PT feedback	Yes	50	18.5	73	27.0	1	1	0.558
	No	37	13.7	110	40.7	2.0 (1.21–3.41)	1.2 (0.54–3.05)	
Does the laboratory undergo root cause analysis	Yes	37	13.7	27	10	1	1	0.321
	No	50	18.5	156	57.8	4.2 (2.37–7.70)	0.5 (0.15–1.83)	
Does the laboratory taking corrective actions for PT nonconformance	Yes	67	24.8	32	11.8	1	1	< 0.001*
	No	20	7.4	151	55.9	15.8 (8.43- 29.6)	7.1 (2.99–16.87)	

*AOR* adjusted odds ratio, *COR* crude odds ratio, *CI* confidence interval, *#* frequency, *%* percentage, *1* reference.

*Statistically significant.

## Discussion

This study focused on assessing the performance of PTs and identifying the determinant factors among comprehensive specialized hospital laboratories in northwest Ethiopia. The findings of this study indicated that for each of the 30 test parameters in the three participating hospital laboratories, the overall failure rate in the laboratory was 32.4%. This finding is greater than 20% compared to the maximum failure rate of the CLIA standard guidelines^15^. These findings are also higher than the 16.5% and 1.63% reported in studies conducted in Ghana and Burkina Faso^16^ and sub-Saharan Africa laboratories^17^. This might be due to low participation, PT samples not being performed because of reagent unavailability and/or equipment failure, not using appropriate units of measurement for the PT result report, or a lack of corrective actions taken for PT nonconformances. However, these findings are lower than those of a study conducted in Addis Ababa, Ethiopia^18^, in which the failure rate was 63.2%. The variations in the number of study years and laboratory test parameters included in this study might be possible reasons for the differences.

The study’s findings indicate that throughout the study years, the overall failure rate gradually dropped from 40.3 to 36.3% and 20.6%, respectively. This could be due to the interventions taken by the laboratory managers and professionals for the gap identified through the PT procedure. In addition, technological advancements might also have an effect on this improvement. Furthermore, participating laboratories undergo interlaboratory discussion between laboratory professionals and the management unit in each cycle for the PT nonconformances if any, which lead to undergoing root cause analysis for the identification of the real cause of the problem and taking corrective actions that prevent the problem from reoccurring, which have a direct effect on the improvement of the PT performances in the proceeding cycles. These findings are comparable to those of a study conducted in Bhutan, in which the prevalence decreased from 37 to 21%^19^, and to those of Addis Ababa, in which the prevalence decreased from 34 to 29.2%^20^. The variation in percentage might be because of variations in the number of study years, participating laboratories, and test parameters.

The average failure rates of PT in microbiology, clinical chemistry, parasitology, hematology, and molecular biology were 56.5%, 37.2%, 29.6%, 29.2%, and 22.2%, respectively. These findings are greater than those of studies conducted in Burkina Faso^21^, in which the average failure rates were 3%, 10%, 13%, 9.3%, and 6%, respectively, for each section of the laboratory. This discrepancy might be due to variations in the biological components, storage conditions, and shipment of PT samples. Differences in equipment, professionals, and infrastructure in the laboratory might also be possible reasons.

The findings of this study suggested that the overall failure rate of laboratory tests gradually decreased in terms of clinical chemistry, microbiology, and molecular biology. However, in hematology and parasitology, the overall failure rate continues to vary from year to year. This might be due to the difficulty of sample management during transportation and shipment. Since whole blood is more labile than plasma and serum, it may deteriorate faster during shipment. On the other hand, parasitological samples such as ova and trophozoites are dead and deteriorate faster.

Based on the multivariable logistic regression, the risk of high failure rates in laboratories with equipment failure and downtime was 2.6 times greater than that in laboratories without equipment failure. These findings are supported by the findings of studies conducted in resource-limited countries^22^. On the other hand, laboratories without adequate reagents are 6.1 times more likely to score a high failure rate than are those with adequate reagents. These findings are supported by the findings of studies conducted in Sub-Saharan Africa and Addis Ababa^17,23^. This could be because a lack of laboratory reagents and equipment failures caused a service interruption, resulting in PT samples not being performed because tests were suspended during PT events, which led to a PT score of zero, which increased the failure rate. In addition, a laboratory without daily IQC practice is 3.8 times more likely to score high failure rates than one with IQC practices. These findings are supported by a study conducted in Ethiopia^20^. This is because performing daily IQC increases the quality of routine laboratory testing. Sustaining routine laboratory test quality increases the overall performance of the laboratory by reducing analytical errors, which reduces the overall failure rate of PT in the laboratory.

Based on the regression analysis of this study, laboratories reporting PT results without the appropriate unit of measurement are 7.5 times more likely to score high failure rates than are those with the appropriate unit of measurement. This might be because even if the PT results are numerically correct, without an appropriate unit of measurement, they will result in an unacceptable PT performance score. Moreover, laboratories not taking corrective action for PT nonconformance are 7.1 times more risky for poor performance than are those taking corrective action. This might be because identifying analytical gaps, undergoing root cause analysis, and taking corrective action are vital for scoring good performance and for continuous quality improvement.

Furthermore, the findings of this study suggest establishing a standardized system for implementing PT programs in each participating laboratory because different factors affecting the implementation of PT, such as not using appropriate units of measurement and not taking corrective actions for PT nonconforming results, might be due to the lack of a standardized system on how to implement and report the PT results, which is vital for the improvement of laboratory PT performance.

## Conclusion

Based on the study’s findings, the overall [performance of the laboratory was lower. But, there is an improvement within the three consecutive years even if it needs further improvement. The failure to remediate PT nonconformances, PT result reports without an appropriate unit of measurement, and the unavailability of reagents were the main causes of the poor performance of the laboratories. In addition, a lack of daily IQC practice and equipment failure and downtime had an impact on PT performance. For certification and ongoing quality improvement, participating laboratories should therefore pay close attention to those elements to improve their performance.

## Supplementary Information

Supplementary Table S1.

## Data Availability

The data used to support the findings of this study are available from the corresponding author upon request.
