# Author Correction: Performances and determinants of proficiency testing in clinical laboratory services at comprehensive specialized hospitals, northwest Ethiopia

**DOI:** 10.1038/s41598-024-84225-2

**Published:** 2025-01-16

**Authors:** Negesse Cherie, Teshiwal Deress, Maereg Wolde, Bisrat Teketelew, Mebratu Tamir, Abiy Angelo, Amare Mekuanint Terekegne, Elias Chane, Mesele Nigus, Dereje Mengesha Berta, Kasaw Adane

**Affiliations:** 1 Department of Quality Assurance and Laboratory Management, School of Biomedical and Laboratory Sciences, College of Medicine and Health Sciences, University of Gondar, Gondar, Ethiopia; 2 Department of Health Promotion and Health Behavior, College of Medicine and Health Science, University of Gondar, Gondar, Ethiopia; 3 Department of Hematology and Immunohematology, School of Biomedical and Laboratory Sciences, College of Medicine and Health Sciences, University of Gondar, Gondar, Ethiopia; 4 Department of Medical Parasitology, School of Biomedical and Laboratory Sciences, College of Medicine and Health Sciences, University of Gondar, Gondar, Ethiopia; 5 Department of Immunology and Molecular Biology, School of Biomedical and Laboratory Sciences, College of Medicine and Health Sciences, University of Gondar, Gondar, Ethiopia; 6 Department of Clinical Chemistry, School of Biomedical and Laboratory Sciences, College of Medicine and Health Sciences, University of Gondar, Gondar, Ethiopia

Correction to: *Scientific Reports* 10.1038/s41598-024-58525-6, published online 02 April 2024

Teshiwal Deress, Maereg Wolde and Kasaw Adane were omitted from the author list in the original version of this Article.

The Author Contributions section now reads:

“NC played a key role in conceptualizing ideas, developing the proposal, overseeing the data collection, data analysis and interpretation, manuscript writing, and communicating its contents. DM and BB actively participated in idea conception, data analysis, and manuscript. writing. MT, AM, AA, MN and EC contributed to idea conception, data cleaning, and manuscript writing. MW contributed to idea conception, data collection, and manuscript writing. KA and TD actively participated in idea conception, proposal development, data collection, and writeup of the manuscript. All the authors have approved the submitted version and agreed to be accountable to ensure that questions related to the accuracy or integrity of any part of the work.”

The original Article has been corrected.

