# Supplementary material for: Performances and determinants of proficiency testing in clinical laboratory services at comprehensive specialized hospitals, northwest Ethiopia
Source: Sci Rep. 2024 Apr 2;14:7745. doi: 10.1038/s41598-024-58525-6 (PMC10987491; doi:10.1038/s41598-024-58525-6)
Supplement: Supplementary file 1 — Supplementary Table S1. [file 41598_2024_58525_MOESM1_ESM.pdf]

Supplementary files

Performances and Determinants of Proficiency Testing in Clinical Laboratory Services at Comprehensive Specialized Hospitals, Northwest Ethiopia

Negesse Cherie[1]*, Bisrat Birke Teketelew[2], Mebratu Tamir[3], Abiy Ayele Angelo[4], Amare Mekuanint Terekegne[5], Elias Chane[5], Mesele Nigus[1], Dereje Mengesha Berta[2]

Table S1: Distribution of laboratory performances for 30 analyte in nine cycles of proficiency testing program carried out during the years 2020 -2022, Northwest Ethiopia

		2020						2021						2022						Commulati ve	
		Cycle1		Cycle2		Cycle 3		Cycle 1		Cycle 2		Cycle3		Cycle 1		Cycle 2		Cycle 3			
Analytes	Status	#	%	#	%	#	%	#	%	#	%	#	%	#	%	#	%	#	%	#	%
	ACC	5	33.3	5	33.3	10	66.6	13	86.6	13	86.6	6	40	15	100	13	86.6	14	93.3	94	69.9
Glucose	UNACC	10	66.6	10	66.6	5	33.3	2	13.3	2	13.3	9	60	0	0	2	13.3	1	6.7	41	30.3
	ACC	5	33.3	15	100	15	100	9	60	8	53.3	3	20	15	100	14	93.3	15	100	99	73.3
GPT	UNACC	10	66.6	0	0	0	0	6	40	7	46.6	12	80	0	0	1	6.6	0	0	36	26.6
	ACC	5	33.3	15	100	15	100	15	100	14	93.3	8	53.3	15	100	15	100	15	100	117	86.6
GOT	UNACC	10	66.6	0	0	0	0	0	0	1	6.6	7	46.6	0	0	0	0	0	0	18	13.3
	ACC	4	26.6	5	33.3	5	33.3	10	66.6	5	33.3	7	46.6	5	33.3	10	66.6	6	40	57	42.2
Albumin	UNACC	11	73.3	10	66.6	10	66.6	5	33.3	10	66.6	8	53.3	10	66.6	5	33.3	9	60	78	57.7
	ACC	9	60	0	0	9	60	5	33.3	14	93.3	10	66.6	14	93.3	5	33.3	7	46.6	73	54
T.protien	UNACC	6	40	15	100	6	40	10	66.6	1	6.6	5	33.3	1	6.6	10	66.6	8	53.3	62	45.9
	ACC	14	93.3	11	73.3	15	100	15	100	14	93.3	15	100	12	80	14	93.3	14	93.3	124	91.8
ALP	UNACC	1	6.6	4	26.7	0	0	0	0	1	6.7	0	0	3	20	1	6.7	1	6.7	11	8.2
	ACC	6	40	4	26.7	0	0	15	100	5	33.3	10	66.7	6	40	5	33.3	10	66.7	61	45.1
Bil.T	UNACC	9	60	11	73.3	15	100	0	0	10	66.7	5	33.3	9	60	10	66.7	5	33.3	74	54.9
Bil.D	ACC	4	26.7	2	13.3	5	33.3	14	93.3	1	6.7	9	60	5	33.3	4	26.7	5	33.3	49	36.2

	UNACC	11	73.3	13	86.7	10	66.7	1	6.7	14	93.3	6	40	10	66.7	11	73.3	10	66.7	86	63.8
	ACC	4	26.7	5	33.3	0	0	8	53.3	5	33.3	5	33.3	15	100	8	53.3	14	93.3	64	47.4
urea	UNACC	11	73.3	10	66.7	15	100	7	46.7	10	66.7	10	66.7	0	0	7 9	46.7	1	6.7	71	52.6
	ACC	9	60	7	46.7	10	66.7	9	60	13	86.7	11		15	100	10	66.7	14	93.3	98	72.6
Creatine	UNACC	6	40	8	53.3	5	33.3	6	40	2	13.3	4	26.7	0	0	5	33.3	1	6.7	37	27.4
	ACC	5	33.3	5	33.3	8	53.3	7	46.7	9	60	12	80	7	46.7	10	66.7	9	60	72	53.3
Chol.T	UNACC	10	66.7	10	66.7	7	46.7	8	53.3	6	40	3	20	8	53.3	5	33.3	6	40	63	46.7
	ACC	13	86.7	9	60	14	93.3	8	53.3	10	66.7	10	66.7	15	100	10	66.7	10	66.7	89	65.9
Tg	UNACC	2	13.3	6	40	1	6.7	7	46.7	5	33.3	5	33.3	0	0	5	33.3	5	33.3	46	34.1
C.morphology	ACC	5	33.3	10	66.7	0	0	7	46.7	0	0	7	46.7	6	40	8	53.3	0	0	43	31.9
	UNACC	10	66.7	5	33.3	15	100	8	53.3	15	100	8	53.3	9	60	7	46.7	15	100	92	68.1
	ACC	15	100	14	93.3	10	66.7	13	86.7	5	33.3	10	66.7	13	86.7	15	100	15	100	100	74.0
WBC	UNACC	0	0	1	6.7	5	33.3	2	13.3	10	66.7	5	33.3	2	13.3	0	0	0	0	35	26.0
	ACC	15	100	10	66.7	10	66.7	14	93.3	5	33.3	10	66.7	14	93.3	15	100	15	100	108	80
RBC	UNACC	0	0	5	33.3	5	33.3	1	6.7	10	66.7	5	33.3	1	6.7	0	0	0	0	27	20
	ACC	15	100	10	66.7	10	66.7	15	100	5	33.3	10	66.7	14	93.3	15	100	15	100	109	80.7
HGB	UNACC	0	0	5	33.3	5	33.3	0	0	10	66.7	5	33.3	1	6	0	0	0	0	26	19.3
	ACC	13	86.7	6	40	9	60	13	86.7	4	26.7	9	60	13	86.7	14	93.3	13	86.7	94	69.6
HCT	UNACC	2	13.3	9	60	6	40	2	13.3	11	73.3	6	40	2	13.3	1	6.7	2	13.3	41	30.4
	ACC	15	100	14	93.3	10	66.7	15	100	5	33.3	10	66.7	15	100	15	100	15	100	114	82.3
PLT	UNACC	0	0	1	6.7	5	33.3	0	0	10	66.7	5	33.3	0	0	0	0	0	0	21	19.7
	ACC	15	100	9	60	10	66.7	15	100	4	26.7	10	66.7	15	100	15	100	15	100	108	80
MCV	UNACC	0	0	6	40	5	33.3	0	0	11	73.3	5	33.3	0	0	0	0	0	0	21	20
	ACC	15	100	14	93.3	10	66.7	15	100	5	33.3	10	66.7	15	100	15	100	15	100	114	82.3
MCH	UNACC	0	0	1	6.7	5	33.3	0	0	10	66.7	5	33.3	0	0	0	0	0	0	21	19.7
	ACC	15	100	10	66.7	10	66.7	10	66.7	5	33.3	0	0	10	66.7	10	66.7	10	66.7	80	59.2
MCHC	UNACC	0	0	5	33.3	5	33.3	5	33.3	10	66.7	15	100	5	33.3	5	33.3	5	33.3	55	41.8
	ACC	14	93.3	10	66.7	10	66.7	15	100	5	33.3	8	53.3	14	93.3	15	100	15	100	106	75.5
RDW	UNACC	1	6.7	5	33.3	5	33.3	0	0	10	66.7	7	46.7	1	6.7	0	0	0	0	29	24.5
B.idfn	ACC	6	40	0	0	7	46.7	13	86.7	9	60	9	60	9	60	12	80	13	86.7	78	57.8

	UNACC	9	60	15	100	8	53.3	2	13.3	6	40	6	40	6	40	3	20	2	13.3	57	42.2
Gram stain	ACC	5	33.3	6	40	10	66.7	5	33.3	12	80	14	93.3	10	66.7	10	66.7	13	86.7	85	63.0
	UNACC	10	66.7	9	60	5	33.3	10	66.7	3	20	1	6.7	5	33.3	5	33.3	2	13.3	50	37.0
AFB stain	ACC	15	100	15	100	14	93.3	13	86.7	7	46.7	13	86.7	14	93.3	15	100	15	100	121	89.6
	UNACC	0	0	0	0	1	6.7	2	13.3	8	53.3	2	13.3	1	6.7	0	0	0	0	14	10.4
Culture	ACC	14	93.3	7	46.7	12	80	11	73.3	8	53.3	5	33.3	10	66.7	9	60	9	60	86	63.7
	UNACC	1	6.7	8	53.3	3	20	4	26.7	7	46.7	10	66.7	5	33.3	6	40	6	40	49	36.3
Gene expert	ACC	5	83.3	2	33.3	5	83.3	4	66.7	6	100	6	100	6	100	6	100	6	100	46	85.2
	UNACC	1	16.7	4	66.7	1	16.7	2	33.3	0	0	0	0	0	0	0	0	0	0	8	14.8
CD4	ACC	2	33.3	2	33.3	4	66.7	4	66.7	2	33.3	2	33.3	2	33.3	3	50	1	16.7	22	40.7
	UNACC	4	66.7	4	66.7	2	33.3	2	33.3	4	66.7	4	66.7	4		3	50	5	83.3	32	59.3
HIV	ACC	15	100	10	66.7	15	100	15	100	10	66.7	15	100	15	100	15	100	15	100	125	92.6
	UNACC	0	0	5	33.3	0	0	0	0	5	33.3	0	0	0	0	0	0	0	0	10	7.4
Blood film	ACC	4	66.7	3	50	5	83.3	4	66.7	3	50	4	66.7	6	100	5	83.3	4	66.7	38	70.4
	UNACC	2	33.3	3	50	1	16.7	2	33.3	3	50	2	33.3	0	0	1	16.7	2	33.3	16	29.6
**Cumulative performance of analytes in the nine cycles**																**ACC**		**2573**		2573/3807 x 100 = **67.58%**	
																**UNACC**		**1234**			
																**Total**		**3807**			
